# Group Augmentation in Realistic Visual-Search Decisions via a Hybrid Brain-Computer Interface

**DOI:** 10.1038/s41598-017-08265-7

**Published:** 2017-08-10

**Authors:** Davide Valeriani, Caterina Cinel, Riccardo Poli

**Affiliations:** Brain Computer Interfaces and Neural Engineering Laboratory, School of Computer Science and Electronic Engineering, University of Essex, Wivenhoe Park, Colchester, CO4 3SQ UK

## Abstract

Groups have increased sensing and cognition capabilities that typically allow them to make better decisions. However, factors such as communication biases and time constraints can lead to less-than-optimal group decisions. In this study, we use a hybrid Brain-Computer Interface (hBCI) to improve the performance of groups undertaking a realistic visual-search task. Our hBCI extracts neural information from EEG signals and combines it with response times to build an estimate of the decision confidence. This is used to weigh individual responses, resulting in improved group decisions. We compare the performance of hBCI-assisted groups with the performance of non-BCI groups using standard majority voting, and non-BCI groups using weighted voting based on reported decision confidence. We also investigate the impact on group performance of a computer-mediated form of communication between members. Results across three experiments suggest that the hBCI provides significant advantages over non-BCI decision methods in all cases. We also found that our form of communication *increases* individual error rates by almost 50% compared to non-communicating observers, which also results in worse group performance. Communication also makes reported confidence uncorrelated with the decision correctness, thereby nullifying its value in weighing votes. In summary, best decisions are achieved by hBCI-assisted, non-communicating groups.

## Introduction

Several decades of research in decision-making have shown that groups usually make better decisions than individuals (wisdom of crowds). This applies to both when groups face complex decisions requiring evaluation of information of different nature, from different sources and, possibly, acquired over an extended period of time^1, 2^, to circumstances in which rapid perceptual judgements have to be made, for example, when estimating uncertain quantities^3^, finding the correct mapping between letters and numbers^4^, or performing memory tasks^5^. In these cases, the augmented capabilities and intelligence achieved by groups are the result of integrating different views and percepts through the interaction of group members. The advantages of groups, however, can be limited, if not nullified, this depending on a large number of factors, including coordination of resources and communication^6^, sharing of information^7^, group style^8^, judgement biases and leadership^6, 9^. For example, in the presence of a leader who is too dominant, collegial decisions may end up being unilateral decisions. Also, sometimes group decisions made by freely-communicating individuals may be worse than the decisions made by the best individual^10, 11^. This is particularly true when there are time constraints on the decisions.

Given the negative impact that the interaction of group members can have on decisions in some circumstances, one may wonder whether we could use technology to obtain the advantages of groups *without* member interactions. It is plausible to think, in fact, that group decisions could be partly based on the integration of neural activity of the group’s members, particularly in circumstances that require rapid decisions. This idea has recently been explored with collaborative Brain-Computer Interfaces (cBCIs) by Eckstein *et al*.^12^, where the brain activity of up to 20 group members asked to discriminate between rapidly presented pictures of cars and faces was aggregated to obtain group decisions. A cBCI with 8 or more observers was more accurate and faster than a single non-BCI user in this visual discrimination task. Similar results were obtained with a Go/NoGo version of the same task^13^. However, cBCI-assisted groups were always inferior to corresponding non-BCI groups.

To address this limitation, we recently developed a *hybrid* BCI (hBCI) that used a combination of EEG neural signals and response times (RTs) to estimate the decision confidence of group members^14^, and, ultimately, the accuracy of each response. The relationship between RTs and decision confidence (or certainty) has been widely described in the literature^15–19^, and while the mechanisms behind this relationship are still not fully understood, it is well known that lower decision confidence and accuracy are associated with longer decision times.

In our hBCI^14^, participants performed a visual-matching task where they had to decide, as rapidly as possible, whether or not two sets of shapes presented in rapid succession were identical. To perform feature selection and parameter identification, we used information on whether each observer’s response was correct or incorrect, on the assumption that observers are on average less confident in erroneous decisions than in correct ones. Indeed, an observer is more likely to give an incorrect response when the perceptual processes leading to the decision do not provide all the necessary information to take the correct decision, hence making the user uncertain. On the other hand, it is reasonable to assume that the confidence with which an observer makes a decision would be high for most of the correct trials. Decision confidence was, therefore, interpreted as the probability of a decision being correct^20^. The hBCI used a trained machine-learning component to build correlates of decision confidence. These were used to weigh the decisions of each group member, which were then combined to obtain a final group decision. Tests conducted on the visual-matching task showed that our hBCI was able to achieve, for the first time, *more accurate decisions than those obtained by traditional non*-*BCI groups* using standard majority rule. Similar results were later achieved with a more demanding visual-search task^21^.

While these results are very encouraging, they are not surprising given that the hBCI estimates and uses the decision confidence to weigh individual decisions. In principle, one could more easily and, perhaps, more accurately ask participants themselves to report their decision confidence. This may have both advantages and disadvantages. On the one hand, due to the noise and unreliability of EEG, it would not be surprising if reported confidence was more reliable than the estimates from the hBCI. On the other hand, research has shown that humans do not always report high values of confidence where their decisions are more likely to be correct and *vice versa* ^22^, e.g., overconfident people may report high values of confidence when they are likely to be wrong^23, 24^.

In our previous work, we did not explore the relative benefits and drawbacks of using the confidence reported by the users instead of the confidence estimated by the hBCI as a mechanism to improve group decision making. Moreover, we did not examine whether our findings would only apply to decisions associated with the simple shapes and colours used previously or would also extend to decision tasks with realistic stimuli. Finally, we did not study whether introducing some form of communication within groups would be an advantage or a hindrance.

The aims of this study were to start addressing these three issues and to investigate the conditions in which hBCI-based group decisions are advantageous or deleterious and why. We carried out three visual-search experiments, where EEG data and RTs were recorded. As in our previous experiments, the neural and behavioral features related to each decision were used to estimate the level of confidence of each observer making that decision. In Experiment 1a, we asked participants to perform a visual-search task using *realistic stimuli* instead of simple shapes, with the aim of testing our hBCI in realistic domains, as well as corroborating (or otherwise) our previous findings. On each trial, participants were briefly presented with a display showing a variable number of penguins and where also a polar bear might be present. Participants’ task was to decide, as rapidly as possible, whether or not they saw the polar bear (which was presented on 25% of trials). In Experiment 1b, stimuli and task were identical to those of Experiment 1a. However, participants were also asked to report their confidence after each decision, which allowed us to compare reported and hBCI-estimated confidence values head-to-head as weights for individual decisions. While we are aware that providing the level of confidence after a decision has been made might be less informative compared to when this is done concurrently with the decision^17^, in Experiment 1b we followed the former approach. This allowed us to keep the procedure used in Experiments 1a and 1b — and presumably also the neural correlates of perceptual and decision processes — identical in the two experiments up until the response. Finally, in Experiment 2 we explored the impact of a controlled form of communication on both group decisions and confidence estimation. In both Experiments 1a and 1b, as in our previous research, participants were *isolated* while making decisions and, so, there was no form of interaction. In Experiment 2, pairs of volunteers undertook the same task as for other two experiments and were allowed to exchange information via a computer-mediated form of communication.

## Methods

### Participants

Three experiments were performed for this study: in Experiments 1a and 1b participants were isolated, while in Experiment 2 participants were randomly paired and were allowed to use a constrained form of communication. Ten healthy volunteers took part in Experiments 1a (4 females, mean age = 28.5 years, SD = 6.0) and 1b (5 females, mean age = 27.4 years, SD = 5.5). Eight pairs of healthy participants (7 females, mean age = 28.1 years, SD = 7.2) participated in Experiment 2. The data of one pair, however, had to be discarded, as the neural data recorded from one member of the pair become very noisy half-way through the experiment due to poor electrode contact. Therefore, we only considered the data recorded from the remaining seven pairs in the analysis of Experiment 2. All participants had normal or corrected-to-normal vision and signed an informed consent form before taking part in the study. The research received ethical approval by UK’s MoD and University of Essex in July 2014. All experiments were performed in accordance with relevant guidelines and regulations.

### Stimuli and Procedure

**Figure 1:**
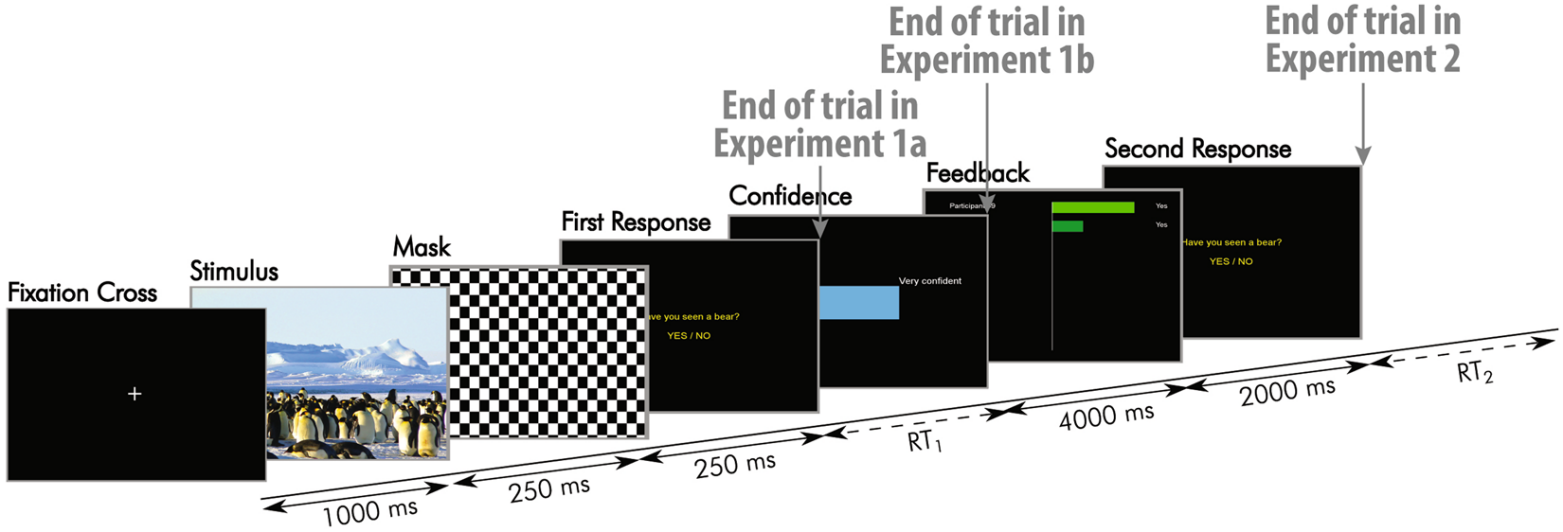
Timeline of a trial. The first four displays were common for all experiments, the fifth display was presented in Experiments 1b and 2, and the last two displays were only presented in Experiment 2.

Each experiment consisted of 8 blocks of 40 trials. In each trial, participants had to decide whether a “target” was present amongst a number of non-targets or “distractors”. Figure 1 shows the timeline of a single trial.

In all experiments, trials started with a fixation cross, displayed for one second. Then an image of an arctic environment was shown for 250 ms. Each picture contained a variable number of penguins (distractors) and possibly a polar bear (target), and was presented fullscreen, subtending approximately 30.3 degrees horizontally and 19.2 degrees vertically. This display was immediately followed by a mask (a black and white 24 × 14 checkerboard) for 250 ms. Participants had to decide, as quickly as possible, whether or not the target was presented, by clicking the left or the right mouse buttons, respectively (1^*st*^ response). RTs were recorded. While this ended a trial of Experiment 1a^25^, the participants of Experiments 1b and 2 were then asked to indicate, within four seconds, the degree of confidence of their decision (0–100%) using the mouse wheel (which varied confidence in 10% steps) – see fifth display in Fig. 1. In Experiment 2, to synchronise participants, a display containing the text “Please wait” was shown to the fastest member of the pair after indicating his/her confidence, until the other member had also indicated his/her confidence. Pair members were then shown a display containing the decisions and the degrees of confidence indicated by each of them for two seconds. This was the only form of communication allowed between the participants. Finally, both pair members were individually asked once again to indicate whether or not the target was present (2^*nd*^ response).

In Experiment 1a, the stimulus set included five images without target and 40 images with the target. The target images were prepared by superimposing to non-target pictures a polar bear chosen from a set of two. Polar bears were positioned in four possible locations. Experiments 1b and 2 used the same images, except that: (1) we discarded six pictures where the average error rate across participants in Experiment 1a was below 10% (too easy) or above 90% (too difficult), and (2) we increased the number of stimuli by including horizontally-flipped versions of the remaining images, resulting in a stimulus set of 68 target images and 10 non-target images.

In each experiment, the same randomly generated sequence of displays was used for all participants. This sequence was identical in Experiments 1b and 2, while in Experiment 1a 17 trials out of 320 were different from those used in the other experiments. Target images occurred in 25% of the trials of each block.

Before an experiment, participants were briefed and familiarised with the task by doing two training blocks of 10 trials each. Preparation and practice took roughly 45 minutes. Then, Experiments 1a, 1b and 2 lasted about 25, 30 and 40 minutes, respectively. Participants controlled the mouse with the preferred hand and were comfortably seated at about 80 cm from an LCD screen. In Experiment 2, participants were randomly paired and tested in different rooms to avoid direct communication.

### Data Recording and Preprocessing

Neural data were acquired from 64 electrode locations according to the international 10–20 system using a BioSemi ActiveTwo EEG system. Each channel was referenced to the average of the electrodes placed on each earlobe. The data were originally sampled at 2048 Hz and then band-pass filtered between 0.15 and 40 Hz with a 14677-tap FIR filter^14^. Artefacts caused by ocular movements were removed by using a standard subtraction algorithm based on correlations to the average of the differences between channels Fp1-F1 and Fp2-F2.

For each trial, stimulus- and response-locked epochs lasting 1900 ms were extracted from the EEG data. The former started 200 ms before the stimulus onset, while the latter started 1200 ms before the participant’s response. The epochs were then detrended and low-pass filtered (pass band of 0–14 Hz and stop band of 16–1024 Hz) with an optimal FIR filter designed with the Remez exchange algorithm. Finally, the data were downsampled to 32 Hz. The first and last 200 ms of each epoch were then trimmed to obtain epochs lasting 1.5 seconds. Each epoch was therefore represented by 48 samples for each channel (i.e., a total of 3,072 values).

RTs were measured by time-stamping the clicks of an ordinary USB mouse.

### Training the hBCI

The main idea behind our hBCI approach to group decision making is assigning higher weights to individual decisions where a participant was confident (i.e., that are likely to be correct) than to those where the participant was not sure. This scenario represents a *well*-*calibrated* system^22^. In order to achieve so, we trained our hBCI system using the *correctness* of individual decisions, as this information is available to the hBCI in the training set. Specifically, the training trials where the decision made by a participant was correct were labelled as “confident” (i.e., label −1), while the trials associated to incorrect decisions were labelled as “nonconfident” (i.e., label +1). With this approach, our hBCI learns to predict whether a user made a correct (confident) or an incorrect (non-confident) decision, instead of predicting whether the response of the user was target or non-target.

A 10-fold cross-validation procedure was used to ensure that the results were not affected by overfitting. In each fold, 288 trials were used as training set and the remaining 32 as test set, to evaluate a group’s performance.

### Confidence Estimation

Common Spatial Pattern (CSP) filtering was used to extract correlates of the decision confidence from the neural data. CSP is a supervised technique which projects the data in a subspace where the variance between two different classes is maximum^26^. Hence, for each participant the trials in the training set were used to compute two transformation matrices, one for stimulus-locked and one for response-locked epochs. These matrices have then been used to transform the neural data collected in each epoch for each trial. The first row of each resulting transformation represented the pattern having the maximum variance. The logarithm of the variance of those was computed and used as neural feature (*nf*). Each trial was therefore represented by two neural features, one extracted from stimulus-locked epochs and one extracted from response-locked epochs.

The RT of the participant in each decision has also been used as an additional behavioural feature, as it correlates with the confidence of the user in that decision^14^.

Logistic regression was then used to predict the confidence weight *w*_*p*,*i*_ (i.e., probability of “correct” class) of the participant *p* in the trial *i* given the feature vector. The logistic regression model was fit using L2 normalisation and a regularisation strength *C* chosen from the set $\{{10}^{-4},{10}^{-3},\ldots ,{10}^{3},{10}^{4}\}$ via stratified cross-validation over the 288 trials of the training set (i.e., in each fold, 192 trials were used for fitting the model and the remaining 96 for validating it with a given *C* value). Logistic loss was used as scoring function for cross-validation.

### Group Decisions

In Experiments 1a and 1b, we formed off-line all possible $$(\begin{array}{c}n\\ m\end{array})$$ groups of size $m=1,\ldots ,10$ with the *n* = 10 participants available. Hence, we had 45 groups of size 2, 120 groups of size 3, etc. For Experiment 2, due to the limited number of identical EEG acquisition devices available in our lab we could not test the effects of concurrent communication on groups larger than pairs. However, we hoped that this experiment could still cast some light on whether the interaction between observers could lead to more accurate *second responses* and, thus, better group decisions. To gain some insight on the performance achievable by larger groups of communicating observers, we combined the available 7 pairs in all possible ways to form groups of size 4, 6, 8 and 10. We chose this way of proceeding instead of the method used in Experiments 1a and 1b to avoid splitting communicating pairs, thereby retaining some of the dynamics observed in such pairs. Hence, we had $$(\begin{array}{c}7\\ 2\end{array})=21$$ groups of size 4, $$(\begin{array}{c}7\\ 3\end{array})=35$$ groups of size 6, and so on. We stopped at groups of size 10 to make results more easily comparable with Experiments 1a and 1b.

For each trial *i*, the decision *G*_*i*_ of a group of *m* observers was obtained by using a weighted majority rule as follows:

1 $${G}_{i}={\mathtt{sign}}\,(\sum _{p=1}^{m}\,{w}_{p,i}\cdot {d}_{p,i}),$$

where *d*_*p*,*i*_ is the decision of participant *p* in trial *i* and ${\mathtt{sign}}$ is the sign operator. So, *G*_*i*_ = +1 if the weighted sum in Equation 1 is positive and *G*_*i*_ = −1 if it is negative. In case of ties (which would produce *G*_*i*_ = 0), a random decision was made between +1 and −1.

In all experiments, we tested the group performance obtained when using traditional groups based on standard majority (i.e., $\forall p$, $\forall i$, *w*_*p*,*i*_ = 1) as well as the performance obtained by groups when the confidence weights were estimated using: (a) only the RTs, (b) only the two neural features, or (c) the RTs and the neural features. Hereafter, we will use the term *RTCI* to refer to the first and the term *nf*-*BCI* to represent the second. We will reserve the acronym *hBCI* to refer to the third type of system, as this is the form of hybrid BCI the article mostly focuses on, albeit also the nf-BCI is a hybrid BCI.

In Experiments 1b and 2 we also tested the use of the subjective confidence indicated by the user in each trial to weigh individual decisions when making group decisions (“Confidence Majority”). For simplicity, we decided to use the reported confidences directly as weights in Equation 1. Naturally, subjective confidence values are likely to include individual biases^22^. However, when we tested (in results not reported) different forms of normalisation for these confidence values (e.g., min-max scaling or division by the median training-set confidence), we found that they did not result in significant improvements of group performance w.r.t. the simpler approach tested here.

Finally, in Experiment 2 we also computed group decisions obtained with standard majority when using the 2^*nd*^ responses provided by participants *after* seeing the decision and the confidence reported by the other group member. We expected these to be more similar to those obtained in traditional interacting groups and, hence, to be more accurate.

## Results

### Confidence-based group decisions are more accurate than traditional ones

**Figure 2:**
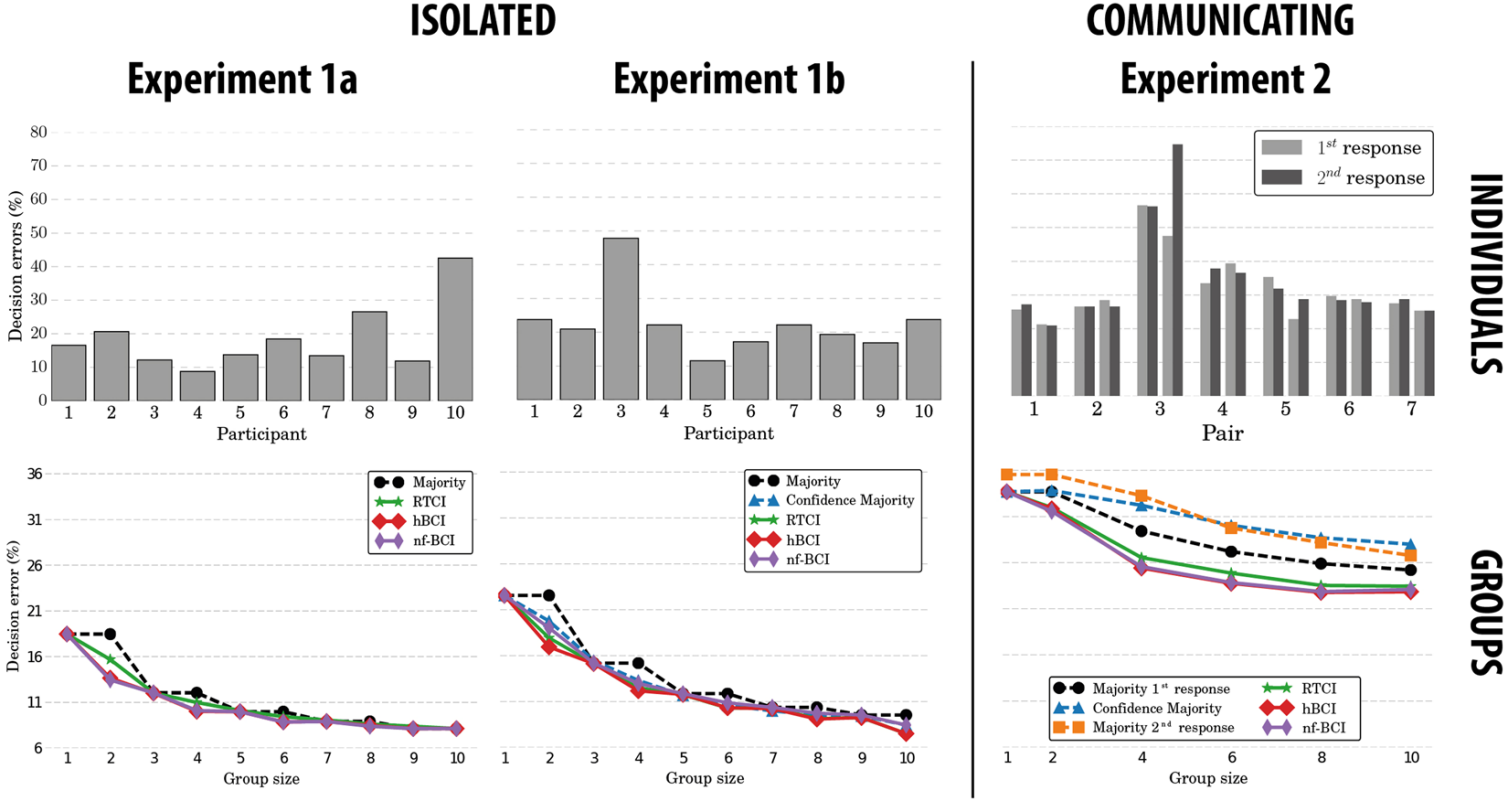
Error rates of each participant (top) and groups of increasing size using different methods (bottom) in the three visual-search experiments in this study. In Experiment 2, individual decision errors are based on either the responses given by observers *before* (light grey) or *after* (dark grey) seeing the decision of the other group member. To preserve the integrity of pairs, in this experiment odd-sized groups were not formed. The average performance of single observers (i.e., groups of size 1) is also reported for reference in the plots at the bottom of the figure.

The decision errors of participants of Experiment 1a are shown in Fig. 2 (top left). The average performance of groups of different sizes using each method is shown in Fig. 2 (bottom left).

From these results it is clear that all confidence-assisted, even-sized groups performed significantly better than majority-based, equally-sized groups — *p* < 0.002 for the one-sided Wilcoxon signed-rank test, *WSR* hereafter. Obviously for groups of size *m* = 10 statistical tests are not possible as we only have one group of that size. In odd-sized groups, hBCI (i.e., the BCI based on neural features and RTs) was able to achieve significant superior performance over all other methods for *m* = 3 (WSR *p* < 0.003) and it was performing on par with majority-based groups for *m* = 5,7,9.

The biggest differences in performance between confidence-assisted and traditional groups occur with groups of an even size because of the intrinsic ability of confidence weights to break ties better than the majority rule (which resolves ties by drawing lots). As a result, adding one member to suitably large, even-sized groups often produces no or marginal performance improvements (*cf*. groups of sizes 4 and 5 in Fig. 2 (bottom left)).

These results confirm the ability of our hBCIs to yield better group decisions in visual search, even with realistic stimuli.

### Neural features and RTs provide complementary information about decision confidence

When comparing the performance of groups assisted by various confidence-based decision systems in Experiment 1a, we found that nf-BCI (which is based only on neural features) was superior to RTCI (based on only RTs) for almost all group sizes (WSR *p* < 0.04 for *m* = 2,4,5,6,7,8 and 9), while the two systems were on par for *m* = 3. Moreover, hBCI (which is based on both neural features and RTs) was also significantly superior to RTCI for almost all group sizes (WSR *p* < 0.003 for *m* = 2,3,4,5,6,7 and 9) and nearly superior for *m* = 8 (WSR *p* = 0.09). When comparing the performance of nf-BCI and hBCI, we found the two methods to be complementary (nf-BCI is superior to hBCI for *m* = 2 and 8, the opposite is true for *m* = 3 and 4, and methods are on par for other group sizes).

In Experiments 1b, hBCI was significantly superior to nf-BCI for all group sizes (WSR *p* < 0.03). It was also superior to RTCI for *m* = 2,4 and 7 (WSR *p* < 0.004), and was not inferior to RTCI for all other group sizes. The small differences in relative advantage of using RT in conjunction with neural features observed in Experiments 1a and 1b are mostly due to small differences in participants behaviours and performance — although individual performance were not significantly different between the experiments (Kruskal-Wallis test, KW hereafter, *p* = 0.11) — as well as the small difference in the protocol used in these two experiments (see Fig. 1).

In Experiment 2, RTCI, nf-BCI and hBCI were on par for *m* = 2, that is for communicating pairs. However, for other group sizes (*m* = 4,6,8 and 10, corresponding to forming groups with 2, 3, 4 and 5 pairs, respectively), hBCI was always significantly superior to RTCI (WSR *p* < 0.005), never inferior to nf-BCI and superior to it for *m* = 10 (WSR *p* < 0.02). It should be noted that in Experiment 2 we have $$(\begin{array}{c}7\\ 5\end{array})=21$$ groups of size 10, unlike in Experiments 1a and 1b where there is one group of that size.

These results show that both the neural features and RTs provide useful information related to the decision confidence but that they also complement each other, hence confirming one of our previous findings^14^. Therefore, in the following sections we will mainly consider the results obtained by the hBCI, as this method achieves the best overall group performance.

### hBCI confidence is slightly superior to reported confidence

Experiment 1a showed that the hBCI can act as a tie-breaker and provide significantly better decisions than standard majority. In Experiment 1b we tested if similar improvements could be achieved by simply asking participants to provide estimates of their confidence after each decision and then using such values to weigh individual responses and make group decisions.

Figure 2 (bottom middle) reports the group performance yielded in Experiment 1b by the four methods already used in Experiment 1a and a weighted-majority method relying on the decision confidence reported by each participant (blue line, labelled “Confidence Majority”). hBCI was significantly better than Confidence Majority for all even-sized groups (*p* < 0.03), was on a par for *m* = 3 and 9 and was significantly worse only for *m* = 5 and 7 (WSR *p* = 0.017). So, hBCI wins over Confidence Majority in 4 cases, draws in 2 and loses in 2. Notably interesting is the superiority of the hBCI for groups of size 2, as these are the most likely to occur in practice.

As one would expect, group decisions made using Confidence Majority were never worse than decisions based on the standard majority rule, being significantly better for group sizes *m* = 2,4,5,6,7 and 8 (WSR *p* < 0.002) and on a par for *m* = 3 and 9. This is only slightly inferior to hBCI, which, as previously indicated, was significantly better than standard majority for all group sizes *m* = 2–9 (WSR *p* < 0.002).

So, while both reported and hBCI-estimated confidence values improve significantly group decisions, the latter presents a slight edge over the former in Experiment 1b.

### Communication worsens individual and group performance

While in Experiments 1a and 1b participants performed their task individually, and their data were later combined into groups of different sizes, in Experiment 2 pairs of observers performed the task concurrently and were allowed to interact using the constrained form of communication described before, which prevented influences of language and body language on results. In these conditions, we were expecting groups to be able to better exploit their augmented perception, hence improving group performance.

The results of Experiment 2 were quite surprising. We expected that based on their *first responses* participants would perform similarly to those of Experiment 1b, as the stimuli and the information available to make a decision were the same. However, taken individually, the first responses of pair members (average error rate = 33.7 ± 10.3%) were 50% worse than those provided by isolated participants (average error rate = 18.5 ± 9.4% and 22.6 ± 9.1% for Experiments 1a and 1b, respectively) — compare Fig. 2 (top left), (top middle) and (top right). Indeed, the error distributions of individual decisions in Experiment 2 before the communication occurred were significantly different from the distributions of isolated observers both in Experiments 1a (KW *p* = 1.57 × 10^−3^) and 1b (KW *p* = 2.55 × 10^−3^).

Even more surprising was the performance obtained when using the *second response*. We expected these responses to be more accurate than the first ones as they integrated the information shared within the pair^3^. However, error rates for individual and pair decisions (obtained with the majority rule) based on these second responses were *not* significantly different from those obtained using the response provided *before* the interaction (two-sided WSR *p* = 0.695). This is illustrated by the values reported for *m* = 1 and *m* = 2 in the black and orange plots in Fig. 2 (bottom right).

While it is known that in certain tasks, such as estimating the number of sweets in a jar^11^ or answering factual questions with a numerical answer^27^, interactions between participants can negatively affect individual performance, we found it surprising that such an effect could occur in the perceptual decision task used in our experiments.

In Fig. 2 (top right) we can see an additional effect of the interaction: in most of the pairs, the performance of the two group members after interaction seems to align, with one participant performing worse in the second decision than in the first and the other participant improving his/her performance. In some cases this slightly increases the average performance of the pair (e.g., pairs 2 and 6), while in others it leads to much worse decisions (e.g., pair 3).

As reported by Lorenz *et al*.^27^, the failures of the wisdom of crowds can be associated with increases in individual confidence when participants are fully aware of other participants decisions. This appears to be happening also in our experiment (more on this later). Indeed pair decisions based on the confidence values reported by the users (blue line in Fig. 2 (bottom right)) were almost as inaccurate (WSR *p* = 0.600) as those obtained by pairs using the majority rule (black line). However, hBCI appeared to be better (on average) than standard majority and Confidence Majority, although the differences did not reach statistical significance (WSR 0.064 < *p* < 0.136). Extrapolating the analysis to larger groups, we found that hBCI is always superior to standard and Confidence Majority (WSR *p* < 10^−4^ for *m* = 4,6,8 and 10). Moreover, decisions made by larger groups using Confidence Majority are significantly worse than those made using standard majority (*p* < 10^−3^ for *m* = 4,6,8 and 10).

### Interaction nullifies the advantages of experience

In Experiment 2, we observed how interaction between individuals can have a detrimental effect on group performance. In this and the following sections we study the causes of such changes.

**Figure 3:**
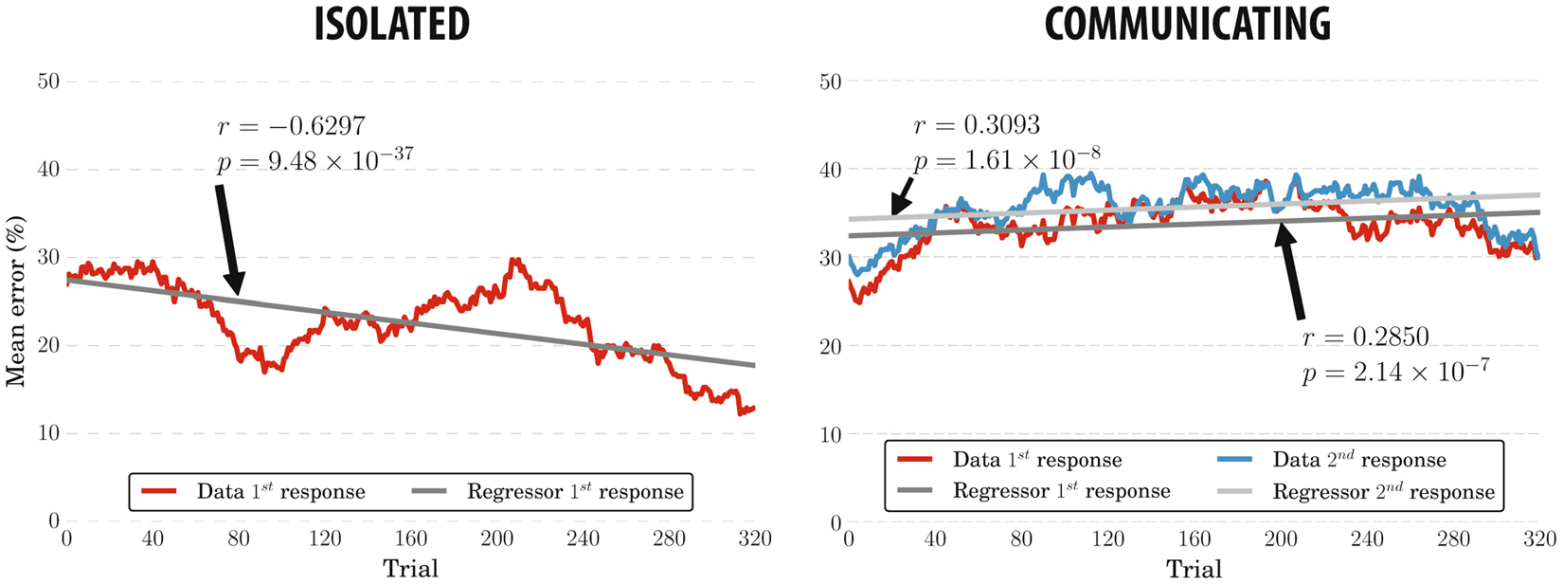
Mean error rates across participants for Experiments 1b (left) and 2 (right) computed using a simple moving average considering 40 consecutive trials. The blue line (right) shows the mean error rates achieved by considering the second response of participants. The grey lines show the linear regressors fit to the data. Their correlation coefficients and two-sided *p*-values are also reported.

Firstly, we analysed how the error rates vary during the experiments. Experience and task familiarisation should improve performance^28^ and, so, we expected higher error rates in the earlier part of an experiment than later on. Indeed, this is what we observed with isolated participants (i.e., in Experiment 1b) – see Fig. 3 (left). Similar results (not reported) were obtained in Experiment 1a. However, with communicating users (i.e., in Experiment 2), surprisingly, we observed the opposite trend, with participants getting worse over time – see Fig. 3 (right). Error rate distributions of Experiments 1b and 2 (shown in red in Fig. 3) were significantly different (KW *p* = 1.39 × 10^−100^).

The average error rates increased slightly when we considered the *second responses* (blue line in Fig. 3 (right)), provided by the participants of the Experiment 2 after seeing the other member’s decision and confidence. The first and second response error rate distributions in Experiment 2 were significantly different (two-sided WSR *p* = 1.44 × 10^−50^).

These results suggest that even the controlled interaction used here negatively affects the individual performance, nullifying the advantages provided by task and group experience. Communicating observers are more cautious^29^ and, therefore, less likely to provide the risky answers based only on gut feelings which are required to perform well in our experiments.

### Communication does not increase the level of agreement nor helps resolve ties

One of the main advantages of groups is their intrinsic error correction capabilities, which could be exploited when the decisions made by their members are *diverse* and observations are *not* correlated^2, 12^. For pairs, this occurs when the observers give different responses, hence generating a tie. We exclude ties as possible outcomes of group decisions. Thus, each voting method for aggregating member decisions must have a tie-breaker strategy and group performance partly depends also on the choice of such strategy.

**Figure 4:**
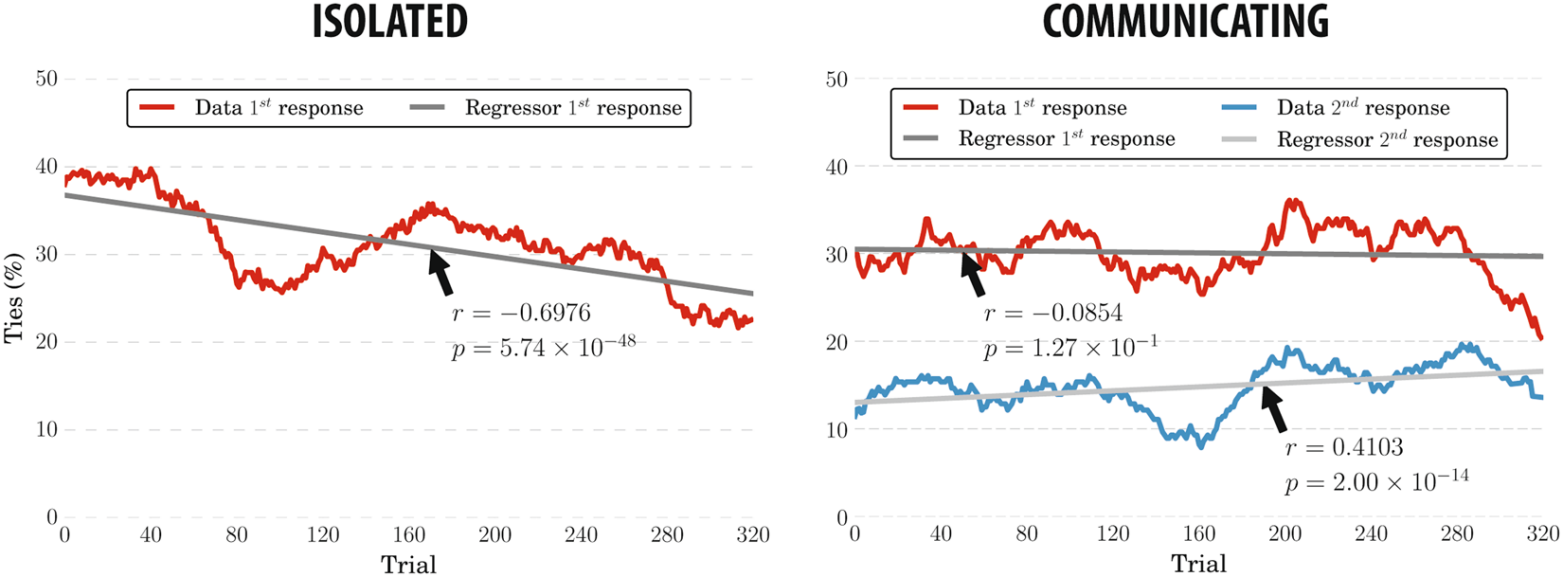
Percentage of trials resulting in a tie, pair members disagreeing on the corresponding decisions (individual points are computed using a moving average across 40 consecutive trials). The values are averaged across the 45 pairs formed with participants of Experiment 1b (left) and the 7 pairs recorded in Experiment 2 (right). The grey lines show the linear regressors fit to the data with their correlation coefficients and two-sided *p*-values.

We analysed how the level of agreement of the pairs varied along Experiments 1b and 2 (Fig. 4). We expected that communicating participants would more likely agree on a decision than non-communicating observers, since the latter have their responses combined into group decisions off-line, without any interaction having taken place.

In Experiment 1b, the number of trials in which the pairs (created off-line) disagree decreases as the experiment progresses. This is reasonable because, as we have seen before (see Fig. 3 (left)), participants performance improved due to the experience and, therefore, the number of ties had to correspondingly reduce through the course of the experiment. However, surprisingly, when observers communicated (Experiment 2), their level of agreement remained almost constant during the experiment – see Fig. 4 (right). Tie dynamics is statistically significantly different between the two experiments (KW *p* = 2.54 × 10^−80^). Partly, this is the result of individual performance not improving over time in Experiment 2 (see previous section).

Experiment 2 gave participants the chance to change their decisions after sharing information about the other member’s response and reported confidence. In these conditions, groups should display increased sensing capabilities and improved cognition. Hence, we expected the level of agreement to be higher and to achieve better performance than that obtained using the decisions provided by the participants before sharing any information. The results shown in Fig. 4 (right) confirmed that the number of ties was much lower when using these second responses. However, as we have seen before (see Fig. 2 (right)), pair performance using the majority method was as bad as that of the first responses.

This can be explained as follows. With majority, we break ties by flipping a coin. Hence, on average, half of the ties result in correct decisions. Given this, if we assume that when participants agree in their first responses they will also agree in the second, then any performance differences between the two conditions can only be the result of any ties in the first response that were resolved by the observers themselves resulting in identical second responses. Since the error rates achieved with the first and second responses are quite similar, it follows that *communication had the same tie*-*breaker performance as coin flipping*.

### Confidence correlates with accuracy only in isolated users

The decision confidence is the probability of the decision to be correct^20^ and, therefore, its value should correlate with the accuracy. Our decision-making systems are based on this assumption. If either the reported or the hBCI confidences are not correlated with the correctness in a decision, this could lead to poor group decisions.

Previous research has shown that group interaction makes individuals more confident on their decisions^27^. However, other studies have not found any systematic difference between the confidence of interacting and isolated decision makers^3^. In the following sections, we verify if the confidences reported by the users or estimated by the hBCI correlate with the accuracy.

We compared the distributions of the behavioural and neural measures used to estimate the decision confidence (i.e., RTs, reported confidence and neural signals) between trials where the observers were correct (*D*_*c*_) and those where they were incorrect (*D*_*i*_). Firstly, we established that the distributions of RTs between *D*_*c*_ and *D*_*i*_ were significantly different in Experiments 1a (KW *p* = 3.8 × 10^−7^), 1b (KW *p* = 9.1 × 10^−24^) and 2 (KW *p* = 5.2 × 10^−5^), thereby confirming that RTs correlate with correctness^14, 15^.

**Figure 5:**
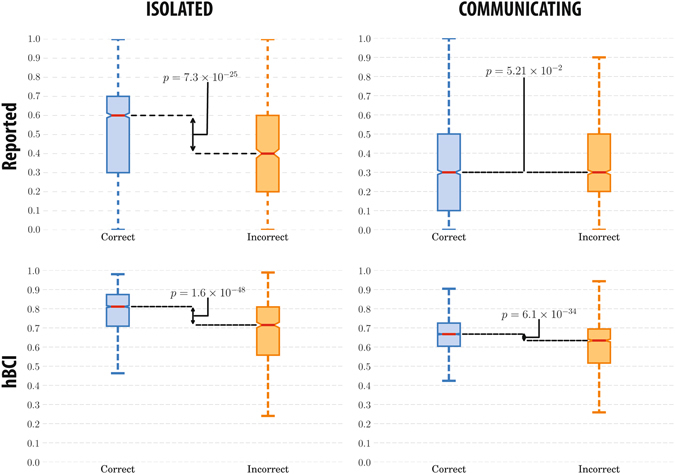
Confidence values indicated by participants (top) and estimated by the hBCI (bottom) for Experiments 1b (left) and 2 (right) for correct and incorrect decisions and corresponding Kruskal-Wallis *p*-values.

We then looked at the reported confidence — see Fig. 5 (top). When participants were isolated (i.e., in Experiment 1b), confidence values were good predictors of the correctness of the decision, as confidence values for *D*_*c*_ and *D*_*i*_ were significantly different (KW *p* = 7.3 × 10^−25^). However, the reported confidences of communicating observers were much less related to the correctness of the first response (KW *p* = 0.052). Nonetheless, the confidence values estimated by our hBCI (Fig. 5 (bottom)) were significantly different for *D*_*c*_ and *D*_*i*_ for both Experiments 1b (KW *p* = 1.6 × 10^−48^) and 2 (KW *p* = 6.1 × 10^−34^), making them good predictors of the correctness of the decision with or without communication.

These results show that reported confidence does not robustly correlate with correctness, as it may be severely influenced by user interactions. When observers are isolated, they report confidence values which are primarily determined by what they have perceived, while when they communicate, their confidence estimates are highly affected by social interactions. Nevertheless, the hBCI confidence is a reliable predictor of the correctness in both conditions, leading to significantly better group decisions.

### Interaction affects neural correlates of decision correctness

Besides participants’ behaviour and performance, we also examined Event Related Potentials (ERPs), as these neural signals are the sources of the features that our hBCI uses to estimate the decision confidence.

**Figure 6:**
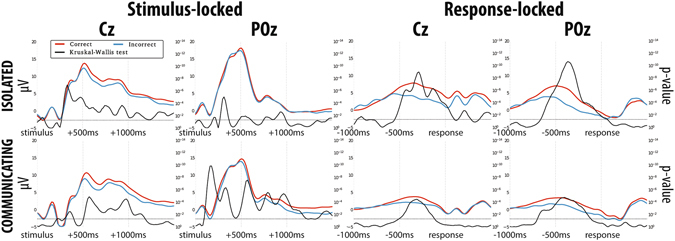
Grand averages of stimulus-locked and response-locked ERPs for the correct (red) and the incorrect (blue) sets and corresponding temporal profile of the KW *p*-values (black) for representative channels Cz and POz for Experiments 1b (first row) and 2 (second row). The horizontal dotted line represents the 5% significance level.

As we did in the previous section, we grouped the ERPs gathered from different participants and trials into two sets, *D*_*c*_ and *D*_*i*_, depending on whether the user made a correct or an incorrect decision, respectively. We then computed the average neural signal (i.e., *grand average*) of each set for Experiments 1b and 2 and we compared the voltages measured at each time step at each electrode location for *D*_*c*_ and *D*_*i*_. Results from two representative electrode sites are shown in Fig. 6.

**Figure 7:**
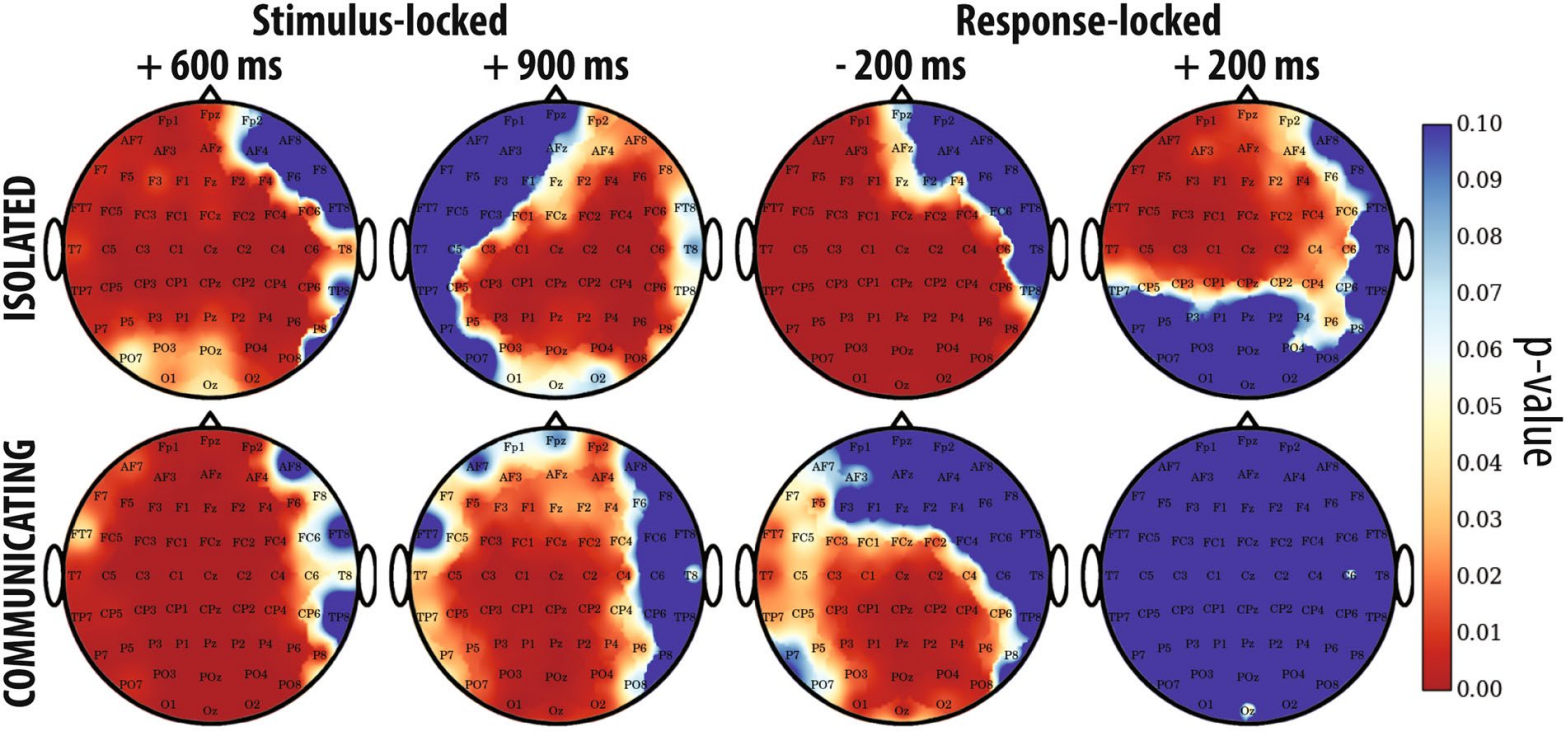
Scalp maps representing the *p* values of the Kruskal-Wallis test used to compare the grand averages of the EEG activity recorded 600 ms and 900 ms after the stimulus onset (first two columns) and 200 ms before and after the response (last two columns) for Experiments 1b (first row) and 2 (second row).

The grand averages of neural signals from both experiments shown in Fig. 6 confirm that ERPs present many statistically significant differences between correct and incorrect trials. These differences exist at many electrode sites, as illustrated in Fig. 7, which shows the scalp maps representing the *p*-values of the KW test used to compare the grand averages for *D*_*c*_ and *D*_*i*_ at critical time steps, for Experiments 1b and 2. We believe that those differences contribute to the ability of the hBCI, illustrated in Fig. 5 (bottom), to provide confidence values that correlate with correctness with both isolated and communicating observers.

We can also see from Fig. 6 that while there are several substantial similarities in the shape between the ERPs recorded from isolated participants (top) and those recorded from communicating participants (bottom), the latter appear to be generally smaller in amplitude. This is true across many other electrodes and times (data not reported). We believe that this most likely reflects the influence that communication and feedback exert on each observer, possibly affecting not only the decision process, but also the perceptual processing itself (see, for example, Balcetis and Lassiter’s and Zadra and Clore’s work^30, 31^).

There is one other marked difference between the ERPs of isolated and communicating observers. This is highlighted by the rightmost plot at the bottom of Fig. 7, which shows a complete lack of statistically significant differences between the ERPs for correct and incorrect trials for communicating observers 200 ms after the response. As illustrated in Fig. 6, this actually starts just before the response and continues for several hundreds of milliseconds after it. This is a clear indication that different cognitive processes are taking place in the minds of communicating observers than in those of isolated ones when they are required to make their decision and report their confidence. These same processes result in much higher error rates — see Fig. 2 (top right) — and much inferior confidence values than for isolated participants, which are also uncorrelated with correctness — see Fig. 5 (top right).

## Discussion

Communication in groups is a double edged sword. It is often a vital means to reach a consensus and optimal decisions, but, it can lead to poor outcomes^6^.

This study presents a hybrid BCI system that can integrate the decisions of multiple observers performing a visual-search task with realistic stimuli *in isolation* and still achieve superior group decisions without the need of any communication. The hBCI uses EEG signals and RTs to estimate the decision confidence of the user, thereby getting a glimpse of the state of the user’s unconscious mind. We have shown that this estimate can act as a tie-breaker, which leads the hBCI to outperform not only the standard majority but also decision-making based on confidence values reported by the participants after each decision.

The proposed hBCI has been applied to a realistic visual-search task in which the average performance of single observers was far from perfect. No improvement over such performance can be obtained by pairs using the majority rule to build group decision if they flip a coin in case of ties. However, by using our hBCI, both pairs and larger groups were able to significantly boost their performance over majority decisions.

This study also analyses the impact of group interaction (via a controlled form of communication) on individual and group performance. When communication within pairs was allowed, users made many more erroneous decisions than when acting in isolation. Moreover, communication had a negative impact on the level of agreement (i.e., the number of ties did not decrease over time, hence requiring a better-than-random tie-breaker, like the hBCI, even more) and on average reported confidence. Furthermore, decisions made by interacting pairs were significantly worse than those made by the average isolated participant. These results suggest that social influence and communication can deteriorate individual and group performance in our visual-search task. Although it was not the purpose of our study to examine the cognitive aspects of decisions of our participants, we suspect that one of the reasons behind the worse performance of communicating observers might be that they trust (or need to trust) their gut feelings less than isolated ones and become less prone to risk than required by the task^29^. Instead, they might be focusing on aligning their behaviour with the one of the other participant.

The changes in the neural signals caused by interaction (the ERPs that characterised correct decisions became more similar to those representing incorrect ones than for isolated participants) made the discrimination between correct and incorrect trials performed by the hBCI more challenging. However, even in these conditions, thanks to its machine learning component which exploits both neural and behavioural features, our hBCI was able to provide a consistent (i.e., results verified with 10-fold cross-validation) and statistically significant improvement in the performance of even-sized groups when compared to traditional groups.

This study has also shown that the confidence reported by the participants after each decision in visual search is an unreliable predictor of correctness. We suggest that this is the result of a series of cognitive processes that are highly influenced by emotions and social interaction. Even though the performance obtained using own confidence estimates with isolated participants was encouraging, in the presence of a constrained form of communication such estimates turned into random predictors of correctness. A possible way of overcoming the negative effects that interaction had in our study, would be providing feedback to each participant about the correctness of their response at the end of each trial. Though this might not completly neutralise the effect of communication, it could make participants more attentive to the task. However, one of the purposes of our study was to simulate decisions in real-world situations, where objective feedback about the correctness of a decision is rarely provided. Therefore, introducing feedback in our experiment would potentially make it less relevant to real-world situations. This, however, might be considered in future research.

To sum up, the results obtained in this study suggest that superior group decisions in visual search are achieved when group members are isolated and their decisions are integrated by using our hBCI based on neural signals and RTs. The confidence estimated by the participants could be a good alternative tie-breaker, but should be used cautiously due to its unpredictable reliability.

In the future, the performance of this hBCI will be tested with tasks other than visual search, including speech perception and face recognition. Moreover, the impact of a more complete form of communication (e.g., including discussion) between participants in pairs and larger groups will be investigated to confirm the findings reported here.

While future research will clarify the scope and benefits of our methods, our hBCI could already be applied to contexts where critical decisions have to be made under time pressure (e.g., defence, health and finance) and where an erroneous decision can cause losses in money or even lives.
